# A compact weak measurement to observe the spin Hall effect of light

**DOI:** 10.1515/nanoph-2023-0675

**Published:** 2023-11-22

**Authors:** Minkyung Kim

**Affiliations:** School of Mechanical Engineering, Gwangju Institute of Science and Technology (GIST), Gwangju 61005, Republic of Korea

**Keywords:** photonic spin Hall effect, precision measurement, Gaussian, splitting, polarization

## Abstract

The spin Hall effect of light (SHEL), a microscopic and transverse splitting of linearly polarized light into circularly polarized components during refraction and reflection, can be measured at subnanometer scales using weak measurements and has emerged as a powerful candidate for precision measurements. However, despite the strong demand for compact and miniaturized sensors and precision metrology, no efforts have downsized the weak measurements. Here I demonstrate that the location of the interface where the SHEL occurs does not impact the results of weak measurements and building on this observation, propose a modified setup called the compact weak measurement to reduce the form factor by replacing one convex lens with a concave one. The concept is theoretically validated and numerically confirmed across various setup parameters and interfaces. The compact weak measurement effectively reduces the required free space distance by twice the focal length and will facilitate the implementation of SHEL-based precision measurements in practical applications.

## 1 Introduction

**Figure 1:**
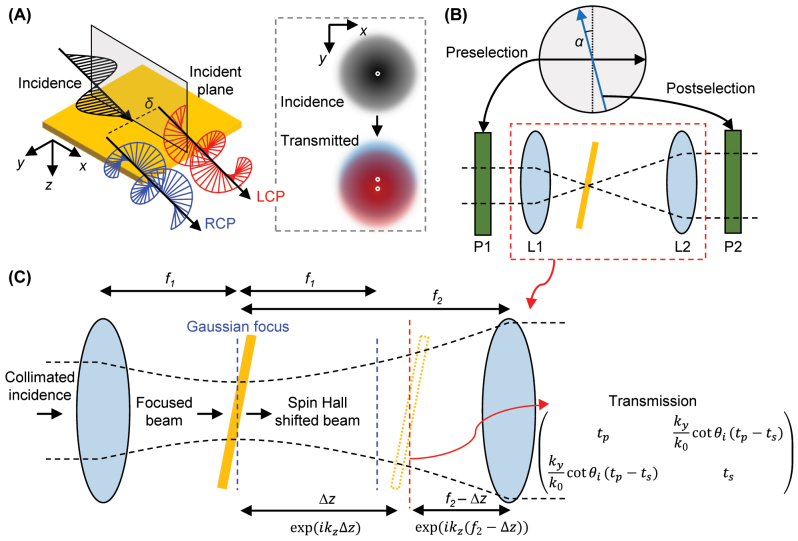
Schematics of the SHEL and weak measurement setup. (A) An illustration of the SHEL. A linearly polarized incidence is split into two opposite circularly polarized components after transmission. The splitting is exaggerated for better visualization. LCP and RCP stand for left and right circular polarizations, respectively. (B) A weak measurement setup to amplify the spin Hall shift. P1 and P2: preselection and postselection polarizers, respectively. L1 and L2: lenses with focal length of *f*_1_ and *f*_2_, respectively. The source and detector are not shown. (C) A magnified view of a portion of the weak measurement setup.

Refraction and reflection, a directional change of light as it propagates through an interface between two different media, are common optical phenomena in our everyday life. The oldest note on refraction and reflection dates back to 300 B.C. when Euclid discovered the law of reflection and described human vision [1]. Refracted or reflected light carries spatial information at the interface and forms a critical basis for scientific achievements from communications [2] to manufacturing [3]. However, one important yet long-standing deficiency in this refraction/reflection process is a transverse splitting of linearly polarized light into two circular polarizations, known as the spin Hall effect of light (SHEL, Figure 1A) [4–9]. The amount of the splitting, also known as the spin Hall shift, is generally subwavelength-scale and has been overlooked for an extended period [10, 11]. Despite its microscopic nature, the spin Hall shift can be a useful measurement target in investigating unknown interfaces because it is determined only by the parameters of the interface and incident beam, such as the Fresnel coefficients, beam waist, wavelength, and incident angle [5, 12, 13]. Therefore, the SHEL has attracted significant scientific attention recently for its applicability to spin-dependent control [14–28] and precision measurements [5, 29–39].

At the core of precision measurements using the SHEL lies the concept of weak measurement [5, 40]. The weak measurement is an experimental method used to measure a quantity corresponding to a small change, called a weak value or weak signal, via preselection and postselection processes and has proven its usefulness in identifying shifts in various quantities such as frequency [41], phase [42], and temperature [43]. A weak measurement setup for the SHEL consists of two pairs of linear polarizers and lenses (Figure 1B) and amplifies the spin Hall shift by several orders of magnitude. Whereas the spin Hall shift at naturally occurring interfaces is generally only a few or a few tens nanometers in the visible, the weak signal, which corresponds to the amplified shift, can reach several hundreds of micrometers, allowing it to be detected using a standard commercial camera detector [30–34]. Because of this selective amplification, the spin Hall shift can be measured with high precision down to the angstrom scale [5] and is considered a potential candidate for sensitively identifying unknown parameters at interfaces. Except that the two linear polarizers can be placed between the lenses and interfaces [5, 44, 45], previous works on weak measurements of SHEL have faithfully followed the weak measurement scheme shown in Figure 1B [46], where the interface, on which the SHEL occurs, is positioned at the focal plane of the lenses. While various efforts have been made to improve the weak measurement and broaden its applicability [47, 48], a compact weak measurement setup to observe the SHEL has yet been proposed despite the strong needs for smaller form factors in industrial implementation and commercialization.

In this paper, I report that the interface where the SHEL occurs is not restricted to be at the focal plane, contrary to our convention, but can be placed anywhere between the two lenses. In other words, the weak signal is independent of the interface position. This independence is straightforwardly explained by the commutative relation between the free space propagation and refraction and is theoretically confirmed by the wave packet model. Furthermore, because of the interface position independence, the two convex lenses in the conventional weak measurement can be replaced with a set of convex and concave lenses, reducing the required free space distance by twice the first focal length. The validity of the compact weak measurement is also proven by numerical simulations using angular spectrum and finite-difference time-domain (FDTD) methods. This compact weak measurement provides a route to investigate unknown interfaces in confined spaces and will contribute to the implementation of SHEL in industries and our daily lives.

## 2 Results and discussion

### 2.1 Concept and principle

A linearly polarized light undergoes a transverse splitting at an interface characterized by Fresnel transmission coefficients *t*_*p*_ and *t*_*s*_ for *p* and *s* polarizations, respectively (Figure 1A), as described in [5]

(1) $$\begin{array}{ll} \delta_{H}^{\pm }/\lambda & =\pm \frac{\cot\theta_{i}}{2\pi }\text{Re}\left(1-\frac{t_{s}}{t_{p}}\right),\\ \delta_{V}^{\pm }/\lambda & =\pm \frac{\cot\theta_{i}}{2\pi }\text{Re}\left(1-\frac{t_{p}}{t_{s}}\right), \end{array}$$

where *δ* represents the spin Hall shift, superscripts + and − denote left and right circular polarizations, respectively, subscripts *H* and *V* correspond to the horizontal and vertical incident polarizations, respectively, *λ* is the wavelength, and *θ*_*i*_ is the incident angle. Eq. (1) holds true when $k_{0}^{2}w_{0}^{2}\gg \cot^{2}\theta_{i}$ , where *k*_0_ = 2*π*/*λ* and *w*_0_ is the beam waist, which is satisfied except for a tightly focused beam under near-normal incidence or with a vanishing Fresnel coefficient. This study only focuses on this large beam waist regime (|*δ*| ≪ *w*_0_) since it is the domain where weak measurement is applicable.

Equation (1) suggests that a straightforward way to achieve a large spin Hall shift is to reduce *θ*_*i*_. However, because the *p* and *s* polarizations are degenerate under normal incidence at the interface between two isotropic media and thus *t*_*p*_ and *t*_*s*_ hardly deviate significantly in their values at small *θ*_*i*_, the spin Hall shift at naturally available interfaces is generally much smaller than the wavelength and the beam spot size. Consequently, the opposite circularly polarized components of the spin Hall shifted beams mostly overlap with each other (Figure 1A, inset) with the shift generally much smaller than the camera pixel size and thus cannot be directly distinguished using a commercial camera detector.

Weak measurement provides an effective solution for quantitatively measuring the subwavelength-scale SHEL by amplifying the spin Hall shift by several orders of magnitude. The weak measurement is composed of two lenses (L1 and L2) and two linear polarizers (P1 and P2) as shown in Figure 1B. A collimated incidence from a laser source is linearly polarized and focused as passing through the first polarizer and lens respectively and then undergoes the SHEL at the interface. The second lens collimates the spin Hall shifted beam and the second polarizer modifies its intensity distribution by filtering out the beam with a spatially inhomogeneous polarization profile into uniformly polarized light. The order of adjacent lens and linear polarizer can be interchanged [5].

The amplification factor through this process is [5, 46, 49]

(2) $$A\equiv \frac{W}{\delta }=F\cot\alpha ,$$

where *W* is the weak signal, *F* = *f*_2_/*z*_*R*_ is the propagation factor, *f*_2_ is the focal length of L2, $z_{R}=k_{0}w_{0}^{2}/2$ is the Rayleigh length of the focused beam, respectively, and *α* is the postselection angle, the angle difference between the postselection polarization and normal of preselection polarization (Figure 1B, top). The amplification originates from two contributions: postselection from two linear polarizers (cot*α*) and propagation effect from two lenses (*F* or more rigorously $\sqrt{1+F^{2}}$ ), each contributing by generally two or three orders of magnitude. Because *w*_0_ = *λf*_1_/*πw*_0*l*_ is determined by the laser beam radius (*w*_0*l*_) and the focal length of L1 (*f*_1_), the second contribution is determined mainly by the focal lengths of two lenses. Therefore, the lenses are selected to provide significant amplification while ensuring that the following condition is satisfied [48]:

(3) $$\frac{\left|\delta \right|}{w_{0}}\ll \min\left(\tan\alpha ,\;\cot\alpha \right),$$

so that the linear relation between the spin Hall shift and weak signal (Eq. (2)) holds.

At first glance, the second contribution looks like a magnification using lenses because the spot size of the spin Hall shifted beam increases by *F*. This may be the reason for placing the interface where SHEL occurs at the focal plane of two lenses in previous works. Interestingly, however, the amplification factor is independent on the interface position (Figure 1C) and the interface can be placed anywhere between the lenses (or two polarizers if the polarizers are between the lenses and interface). In short, as long as the interface is between the pairs of polarizer and lens, Eq. (2) holds. Whereas *δ* has already been known to be independent of the angular profile of the incidence in the large beam waist regime (|*δ*| ≪ *w*_0_), to the best of my knowledge, the independence of *A* and, accordingly, that of *W* are unveiled firstly in this paper.

This independence can be straightforwardly explained by the commutative relation of free space propagation and transmission (Figure 1C). The free space propagation by the distance of *d* corresponds to the multiplication by exp(*ik*_*z*_ *d*) where $k_{z}=\sqrt{k_{0}^{2}-k_{x}^{2}-k_{y}^{2}}$ . Meanwhile, transmission is considered by multiplying the matrix [50],

(4) $$\left(\begin{array}{cc} t_{p} & \frac{k_{y}}{k_{0}}\cot\theta_{i}\left(t_{p}-t_{s}\right)\\ \frac{k_{y}}{k_{0}}\cot\theta_{i}\left(t_{p}-t_{s}\right) & t_{s} \end{array}\right).$$

A weak measurement setup where the interface is misaligned from the lens focal plane by △*z* is considered. Then the beam in this weak measurement propagates the free space by △*z*, transmits the interface, and propagates again the free space by *f*_2_ − △*z* in order (Figure 1C). It can be expressed as

(5) $$\begin{array}{ll} \psi_{f} & =\exp\left(ik_{z}\left(f_{2}-△\;z\right)\right)\\ & \;\times \left(\begin{array}{cc} t_{p} & \frac{k_{y}}{k_{0}}\cot\theta_{i}\left(t_{p}-t_{s}\right)\\ \frac{k_{y}}{k_{0}}\cot\theta_{i}\left(t_{p}-t_{s}\right) & t_{s} \end{array}\right)\\ & \;\times \exp\left(ik_{z}△\;z\right)\psi_{i}, \end{array}$$

where *ψ*_*i*_ is the initial state. As three multiplications in the wave vector space (Eq. (5)) are commutative, △*z* is an irrelavant variable to the final state,

(6) $$\psi_{f}=\exp\left(ik_{z}f_{2}\right)\left(\begin{array}{cc} t_{p} & \frac{k_{y}}{k_{0}}\cot\theta_{i}\left(t_{p}-t_{s}\right)\\ \frac{k_{y}}{k_{0}}\cot\theta_{i}\left(t_{p}-t_{s}\right) & t_{s} \end{array}\right)\psi_{i}.$$

This indicates that the position of the interface has no impact on the final results of the weak measurement and the interface can be placed away from the focal plane as long as it is between the pairs of lens and polarizers.

Another important fact that underlies the interface position independence is that *δ* is independent on the beam spot size on the interface (Eq. (1)) in this large beam waist regime (|*δ*| ≪ *w*_0_). In other words, placing the interface at *z* = △*z* changes the incident spot size but does not affect *δ*. Note that *δ* may vary with the position of interface beyond the large beam waist regime, but such variations are inconsequential as the weak measurement method is not applicable beyond this regime.

### 2.2 Theoretical proof

A rigorous proof of the △*z* dependence in weak measurement is provided in the following using the wave packet model. The beam incident on the interface at *z* = △*z* is described by

(7) $$\psi_{i}=\left(\begin{array}{c} E_{i}^{H}\\ E_{i}^{V} \end{array}\right)\exp\left[-\frac{k_{0}}{2}\frac{x^{2}+y^{2}}{z_{R}+i△\;z}\right],$$

where $E_{i}^{H}$ and $E_{i}^{V}$ represent horizontally and vertically polarized components. Because both the free space propagation and transmission correspond to the multiplication in the wave vector space, the Fourier transform of Eq. (7) is obtained as

(8) $$\begin{array}{ll} F\left[\psi_{i}\right] & =\left(\begin{array}{c} E_{i}^{H}\\ E_{i}^{V} \end{array}\right)\sqrt{\frac{2\pi \left(z_{R}+i△\;z\right)}{k_{0}}}\\ & \;\times \exp\left(-\frac{z_{R}+i△\;z}{2k_{0}}\left(k_{x}^{2}+k_{y}^{2}\right)\right). \end{array}$$

Then the Jones vector of the transmitted beam is

(9) $$\left(\begin{array}{c} E_{t}^{H}\\ E_{t}^{V} \end{array}\right)=\left(\begin{array}{cc} t_{p} & \frac{k_{y}}{k_{0}}\cot\theta_{i}\left(t_{p}-t_{s}\right)\\ \frac{k_{y}}{k_{0}}\cot\theta_{i}\left(t_{p}-t_{s}\right) & t_{s} \end{array}\right)F\left[\psi_{i}\right].$$

To estimate the weak signal under horizontally polarized incidence, I substitute $E_{i}^{H}=1$ and $E_{i}^{V}=0$ , use $t_{p,s}=t_{p,s}\left(k_{x}=0\right)+{\dot{t}}_{p,s}k_{x}/k_{0}$ , where dot denotes the derivative with respect to *θ*_*i*_, to include the finite thickness of the wave packet along the *k*_*x*_-axis, and apply the inverse Fourier transform. Then the transmitted beam represented in the circular polarization basis is

(10) $$\begin{array}{ll} E_{t}^{\pm } & =\exp\left(-\frac{k_{0}}{2}\frac{x^{2}+y^{2}}{z_{R}+i△\;z}\right)\left(t_{p}+i\frac{x}{z_{R}+i△\;z}{\dot{t}}_{p}\right.\\ & \;\left.\pm \frac{y\cot\theta_{i}}{z_{R}+i△\;z}\left(t_{p}-t_{s}\right)\pm i\frac{xy\cot\theta_{i}}{{\left(z_{R}+i△\;z\right)}^{2}}\left({\dot{t}}_{p}-{\dot{t}}_{s}\right)\right). \end{array}$$

Here the constant terms are irrelevant to the final results and omitted here. Remaining before being captured by a detector is the free space propagation by *f*_2_ − △*z*, which can be accounted by adding *f*_2_ − △*z* to △*z* (detailed proof can be found in Supporting Information S1). Then the transmitted beam imaged by the detector is

(11) $$\begin{array}{ll} E_{t}^{\pm } & =\exp\left(-\frac{k_{0}}{2}\frac{x^{2}+y^{2}}{z_{R}+if_{2}}\right)\left(t_{p}+i\frac{x}{z_{R}+if_{2}}{\dot{t}}_{p}\right.\\ & \;\left.\pm \frac{y\cot\theta_{i}}{z_{R}+if_{2}}\left(t_{p}-t_{s}\right)\pm i\frac{xy\cot\theta_{i}}{{\left(z_{R}+if_{2}\right)}^{2}}\left({\dot{t}}_{p}-{\dot{t}}_{s}\right)\right), \end{array}$$

which is equivalent to the previously known formula when the interface is at the Gaussian focus. This process reduces to the derivation of the spatial profiles of the spin Hall shifted beam in the conventional weak measurement by setting △*z* = 0 and also can be proven effortlessly by replacing *z*_*R*_ with *z*_*R*_ + *i*△*z*. Eq. (11) manifests that the position of the interface does not affect the results of the weak measurement as long as it is placed between the two lenses.

### 2.3 Scheme of the compact weak measurement

**Figure 2:**
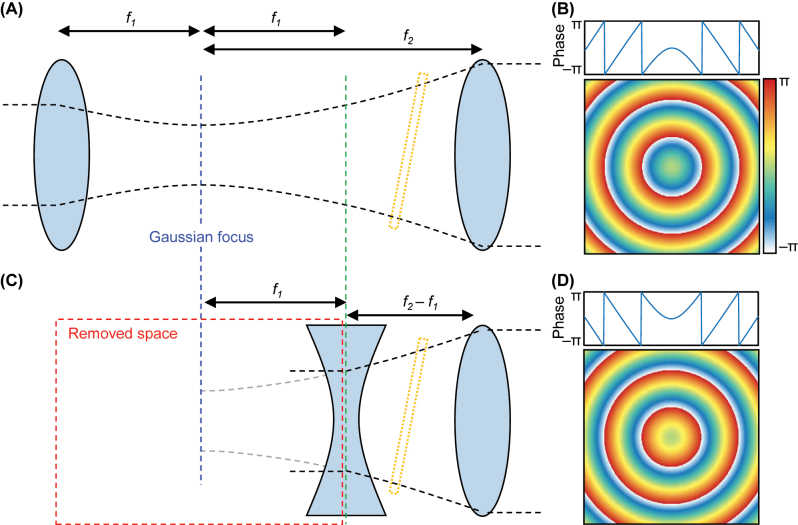
Replacement of the first lens with a concave lens with an equal focal length for compactness. (A) Conventional weak measurement and (B) phase profile of the first lens. (C) The compact weak measurement setup using a set of concave and convex lenses and (D) phase profile of the concave lens. Red box in (C) indicates the removed space by replacing the first lens with the concave lens.

In addition to reducing the need for precise interface positioning, another crucial discovery arising from this independence is the potential removal of a large free space volume within the weak measurement setup (Figure 2). The key principle behind the lens-based amplification is to reduce the Rayleigh length [39, 46] (See Supporting Information S2 for details). The convex lens L1 (Figure 2A) that has the phase profile of $\phi_{\text{conv}}\left(x,y\right)=-k_{0}\left(\sqrt{x^{2}+y^{2}+f_{1}^{2}}-f_{1}\right)$ (Figure 2B) is one but not the only solution for this purpose. A concave lens with the opposite phase profile, $\phi_{\text{conc}}\left(x,y\right)=k_{0}\left(\sqrt{x^{2}+y^{2}+f_{1}^{2}}-f_{1}\right)$ , transforms the incident collimated beam into a Gaussian with the same Rayleigh length as L1 (Figure 2C and D). Specifically, a Gaussian passing through the convex lens and propagating by 2*f*_1_ is equal to the Gaussian right after the concave lens (green dashed in Figure 2A and C). Proof can be found in Supporting Information Section S3. Therefore, one can replace the first convex lens with a concave lens with the same focal length, place it by matching its focal spot with that of L2, and position the interface between the concave lens and L2 (Figure 2C). A weak signal obtained by this compact weak measurement is equal to that of the conventional weak measurement because of the interface position independence.

Recently, space compressors, also known as spaceplates, have been proposed to compress free space distances by emulating the optical transfer function of thicker free space within thinner artificial structures with strong nonlocality [51–57]. While the concept of space compressors holds promise for reducing device size, its use in precise optical setups remains challenging due to incomplete mimicry. In contrast, the compact weak measurement can reduce the required free space distance for measuring the SHEL from *f*_1_ + *f*_2_ to *f*_2_ − *f*_1_ without any distortion or quality degradation. While this compact weak measurement is applicable only when *f*_1_ ≤ *f*_2_, considering that small *f*_1_ and large *f*_2_ are favorable for achieving high amplification $\left(F\propto f_{2}/f_{1}^{2}\right)$ and generally *f*_1_ ≤ *f*_2_ [5, 30–33], the compact weak measurement will be useful in many instances where the SHEL should be measured precisely.

### 2.4 Numerical confirmation using the angular spectrum method

To numerically demonstrate the compact weak measurement, angular spectrum method is used. First, a horizontally polarized Gaussian beam is considered:

(12) $$\psi_{i}=\left(\begin{array}{c} 1\\ 0 \end{array}\right)\exp\left[-\frac{k_{0}}{2}\frac{x^{2}+y^{2}}{z_{Rl}}\right],$$

where $z_{Rl}=k_{0}w_{0l}^{2}/2$ is the Rayleigh length of the beam from the laser. A beam transmitted through the concave lens with a focal length of *f*_1_ is

(13) $$\psi_{L1}=\left(\begin{array}{c} 1\\ 0 \end{array}\right)\exp\left[-\frac{k_{0}}{2}\frac{x^{2}+y^{2}}{z_{Rl}}\right]\exp\left(i\phi_{\text{conc}}\right).$$

Then, the free space propagation by the distance of *f*_2_ − *f*_1_ is included using the angular spectrum method,

(14) $$\psi_{L2}=F^{-1}\left[F\left[\psi_{L1}\exp\left(ik_{z}\left(f_{2}-f_{1}\right)\right)\right]\right].$$

Finally, the postselected beam is obtained by multiplying the corresponding Jones matrix with *ψ*_*L*2_ as

(15) $$\psi_{P2}=\left(\begin{array}{cc} \sin^{2}\alpha & \cos\alpha \sin\alpha \\ \cos\alpha \sin\alpha & \cos^{2}\alpha \end{array}\right)\psi_{L2},$$

and the weak signal *W* is calculated by taking *y* position average of this beam as [5]

(16) $$W=\frac{\iint y|\psi_{P2}{|}^{2}dxdy}{\iint |\psi_{P2}{|}^{2}dxdy}.$$

**Figure 3:**
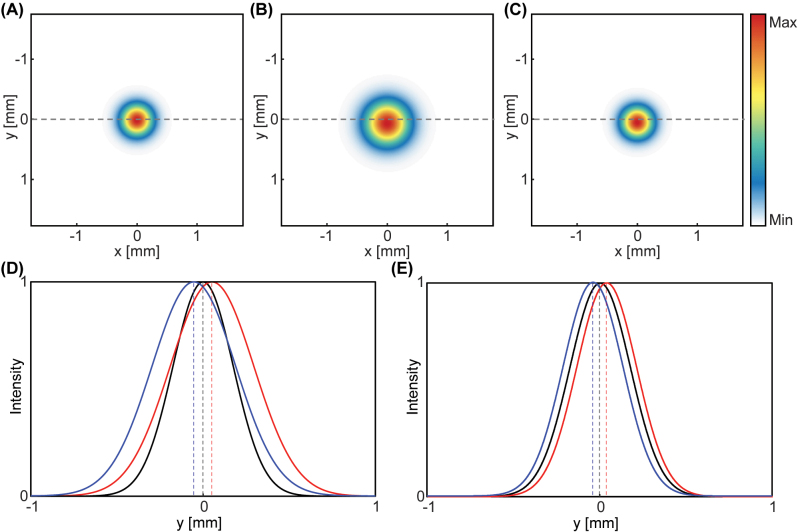
Spatial profiles of the postselected beam obtained using the angular spectrum method. (A) Incident intensity profile. (B, C) Transmitted intensity profiles when *f*_1_ = 25 mm and (B) *f*_2_ = 35 mm and (C) *f*_2_ = 25 mm. (D, E) Cross-sectional intensity profile along the *y*-axis of (B) and (C) respectively. Black: incident, red: postselected with *α* = 1°, blue: postselected with *α* = −1°. All intensities are normalized to have a unity maximum.

Parameters used for the numerical simulation are given as: *λ* = 632 nm, *w*_0*l*_ = 350 µm, *θ*_*i*_ = 60°, *α* = 1°, and *f*_1_ = 25 mm. The interface is a 300 nm-thick slab with the refractive index of 2.5 placed in air. The SHEL at this interface is 27.15 nm, which corresponds to 4.29 × 10^−2^ *λ* (Eq. (1)). The incidence is initially centered at the origin (Figure 3A) and is converted into a diverging beam using the concave lens, undergoes the SHEL, and then is collimated again using the convex lens (Figure 2C). The postselected beam profile when *f*_2_ = 35 mm and *f*_2_ = 25 mm are shown in Figure 3B and C, respectively. The beam radius of the postselected beam is approximately *f*_2_/*f*_1_ of that of the incidence. For the former case, the required free space distance in this compact weak measurement setup is *f*_2_ − *f*_1_ = 10 mm whereas it should be *f*_1_ + *f*_2_ = 60 mm in the conventional setup. Despite of the one-sixth reduced distance, *W* calculated using Eq. (16) (52.20 µm) agrees well with the analytic value, 52.97 µm (Eq. (2)). The slight discrepancy between two values originates from insufficiently small |*δ*|/*w*_0_ ≈ 0.11 tan*α* (Eq. (3)) and becomes smaller than the spatial grid size by increasing *α* so that Eq. (3) is satisfied. The cross-sectional intensity of the postselected beam manifests the amplification clearly (Figure 3D). The displacement occurs along the opposite direction for negative postselection angle.

Meanwhile, when *f*_2_ = *f*_1_, theoretically, no additional free space is required and the minimal distance for placing the set of concave and convex lenses is sufficient (Figure 3C). Whereas the beam radius of the postselected beam is equal to that of the incidence (Figure 3E), the spin Hall shift is amplified by approximately 1.39 × 10^3^. The numerically calculated *W* is 37.71 µm, which shows an excellent agreement with the analytical one, 37.85 µm. This shows that the compact weak measurement amplifies the SHEL into a micrometer scale so that it can be measured in commercially available detectors, but in a much reduced distance that corresponds to (*f*_2_ − *f*_1_)/(*f*_2_ + *f*_1_) of the originally required distance. Note that the weak measurement setup parameters are chosen randomly and are not optimized for the best amplification. The precision of the weak measurement can be further enhanced by optimizing the setup parameters.

**Figure 4:**
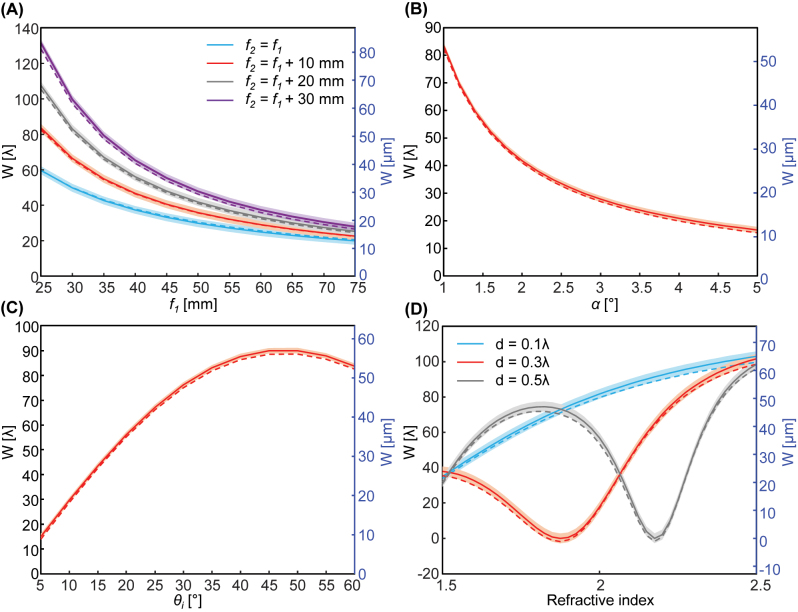
Weak signals under various (A) focal lengths, (B) postselection angle *α*, (C) incident angle *θ*_*i*_, and (D) refractive index and thickness of the slab. Solid: analytic (Eq. (2)), dashed: calculated (Eq. (16)), and shaded: spatial grid size.

To prove the universality of the compact weak measurement, numerically calculated and analytic *W* are compared for various focal lengths, postselection angle, incident angle, and thickness and refractive index of the slab (Figure 4). First of all, *W* is calculated by varying *f*_1_ from 25 mm to 75 mm and setting *f*_2_ − *f*_1_ as 0, 10, 20, 30 mm while remaining other parameters constant (Figure 4A). Solid and dashed curves represent analytical (Eq. (2)) and numerically obtained (Eq. (16)) values respectively and shaded areas denote possible error ranges due to the finite spatial grid size. Because the only variables involved are the focal lengths, the spin Hall shift remains constant, while the alteration in *W* is a result of the change of *A*. The numerical *W* shows the expected tendency $W\propto f_{2}/f_{1}^{2}$ and is within the error bound of the analytical *W* in all parameter space. Figure 4A also demonstrates that increasing *f*_2_ results in a larger *W*, which is advantageous for achieving better precision.

The compact weak measurement also reproduces analytically expected *W* for various postselection angles (Figure 4B) and incident angles (Figure 4C). Other parameters are same as those in Figure 3. Furthermore, *W* is examined at different interfaces by varying the refractive index and thickness of the slab (Figure 4D). Here *A* is a constant because *f*_1_, *f*_2_, and *α* are fixed and only *δ* changes. The compact weak measurement results in *W* that matches well with the theoretical one for all parameter space. This demonstrates the utility of this modified setup for investigating SHEL at various interfaces with different setup parameters.

### 2.5 Numerical confirmation using the FDTD method

**Figure 5:**
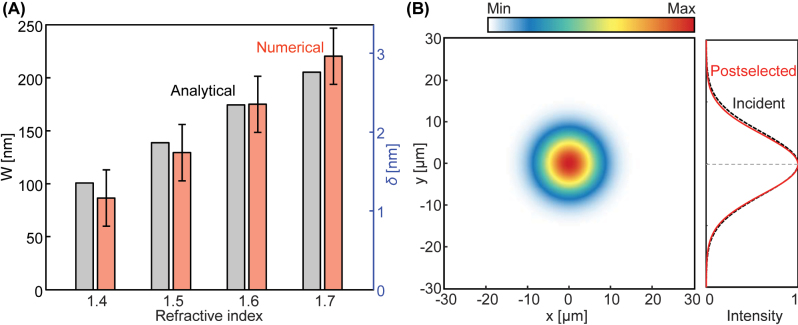
Verification of the compact weak measurement using a full-wave simulation. (A) Analytical (gray) and numerically obtained (orange) weak signals and spin Hall shift for various refractive indices. (B) Spatial profiles of the incident (black) and postselected (red) beams when the index is 1.5.

Finally, the compact weak measurement is confirmed using a full-wave simulation by using a FDTD-based commercial software, Lumerical FDTD (Figure 5). To reduce the memory requirement, different setup parameters are used: *f*_1_ = 250 µm, *f*_2_ = 258 µm, *w*_0*l*_ = 10*λ*, *θ*_*i*_ = 5°. Other parameters are used as the previous. The interface is a slab with a thickness of 100 nm and index varying from 1.4 to 1.7 with 0.1 step size. Numerically simulating the weak measurement using these setup parameters in the conventional way is computationally expensive and challenging as it necessitates a vertical dimension that exceeds 500 µm. In contrast, for this compact weak measurement setup, the simulation domain has a lateral dimension of 80 µm and a vertical dimension of 20 µm. Perfectly matched layer conditions are applied for all boundaries and a uniform mesh with a size of approximately 26.6 nm (≈λ/24) is used. For a Gaussian source, the distance from the Gaussian focus is defined as *f*_1_. This configuration corresponds to the role of the concave lens in the compact weak measurement setup. Then a monitor is placed 8 µm away from the source, where the second lens is supposed to be. A slab is inserted between the source and the monitor. In short, the Gaussian beam from the source passes through the slab and is captured by the monitor. The obtained field profile corresponds to *ψ*_*L*2_ (Eq. (14)). Thus I multiply it by the Jones matrix of the postselection polarizer (Eq. (15)) and average the *y* positions of the postselected beam (Eq. (16)). Because *δ* is a few nanometers at this isotropic-isotropic interface with a small incident angle, the numerically obtained *W* is also small, reaching only a few hundred nanometers (Figure 5). Despite this nanometer scale, it exhibits nice agreements with the theoretical values and the numerical errors are all smaller than the mesh size. The cross-sectional intensity profile when the refractive index of the slab is 1.5 manifests that the postselected beam is shifted from the incidence (Figure 5B).

## 3 Conclusions

In conclusion, I report for the first time that results of weak measurements are independent of the position of interface where SHEL occurs and suggest a compact weak measurement by replacing one convex lens with a concave lens with the same focal length. The interface position independence and the compact weak measurement are confirmed both theoretically and numerically across various parameter spaces. Although this study primarily focuses on the transmission type, the interface position independence also holds for the reflection type and consequently this modified setup can be applied to observe the SHEL of the reflected beam. The compact weak measurement reduces the required free space volume without compromising precision and will be helpful in implementing SHEL-based precision measurements and sensors in practical applications. Finally, while this analysis focuses on the weak measurement for the SHEL using classical optics, follow-up studies to relax setup conditions of other weak measurements, either in classical or quantum regimes, would hold great promise.

## Supplementary Material

Supplementary Material Details
