# Supplementary material for: A compact weak measurement to observe the spin Hall effect of light
Source: Nanophotonics. 2023 Nov 22;12(24):4519–28. doi: 10.1515/nanoph-2023-0675 (PMC11501521; doi:10.1515/nanoph-2023-0675)
Supplement: Supplementary file 1 — Supplementary Material Details [file j_nanoph-2023-0675_suppl_001.pdf]

Supporting Information of
"A compact weak measurement to observe the spin Hall effect of light"

Minkyung Kim[1*]

[1]School of Mechanical Engineering, Gwangju Institute of Science and Technology (GIST), Gwangju 61005, Republic of Korea

[*]m.kim@gist.ac.kr

S1 Free space propagation operator in the real space

This section proves that the free space propagation by d is equivalent to replacing z with $z + d$. Fourier transform of general Gaussian (Eq. S7) satisfies

$$\mathcal{F}[\psi(x, y;\ z_R, z)] \propto \exp\left(-\frac{z_R + iz}{2k_0}(k_x^2 + k_y^2)\right). \tag{S1}$$

The beam after propagating the free space by d can be obtained by multiplying $\exp(ik_z d)$ to the Fourier transformed field as

$$\begin{aligned}\tilde{\psi}_p &= \exp\left(-\frac{z_R + iz}{2k_0}(k_x^2 + k_y^2)\right)\exp(ik_z d)\\ &\approx \exp(ik_0 d)\exp\left(-\frac{z_R + i(z+d)}{2k_0}(k_x^2 + k_y^2)\right),\end{aligned} \tag{S2}$$

where the second line is obtained by applying the Taylor expansion $k_z = \sqrt{k_0^2 - k_x^2 - k_y^2} \approx k_0 - (k_x^2 + k_y^2)/2$. Note that this approximation holds when $k_x^2 + k_y^2 \approx 0$, which is true considering that the beam is not tightly focused and has a narrow wave vector profile. Inverse Fourier transform of Eq. S2 is $\psi(x, y;\ z_R, z + d)$, where the full formula of ψ can be found in section S3, demonstrating that one obtain a Gaussian beam after free space propagation by adding the propagation distance to the last argument in the conventional Gaussian beam profile.

S2 Derivation of the linear relation between the weak signal and propagation factor

This section proves the linear relation between the weak signal and propagation factor $W \propto F = z/z_R$. The spin Hall shifted beam is an overlap of two circularly polarized beams with the opposite

displacement $\pm D$,

$$\psi_{\text{shifted}} = \frac{1}{\sqrt{2}} \sum_{\sigma=\{-1,1\}} \frac{1}{\sqrt{2}} \begin{pmatrix} 1 \\ \sigma i \end{pmatrix} \exp\left(-\frac{k_0}{2} \frac{x^2 + (y - \sigma D)^2}{z_R + iz}\right). \tag{S3}$$

Note that here D is a complex scalar whose real part is equal to the spin Hall shift, $\mathrm{Re}(D) = \delta$. The postselected beam can be obtained by applying the inner product of postselection polarization $\begin{pmatrix} \sin\alpha & \cos\alpha \end{pmatrix}$ as

$$\begin{aligned}
\psi_{\text{postselected}} &= \frac{1}{2} \sum_{\sigma=\{-1,1\}} (\sin\alpha + i\sigma\cos\alpha) \exp\left(-\frac{k_0}{2} \frac{x^2 + (y - \sigma D)^2}{z_R + iz}\right) \\
&= \frac{1}{2} \exp\left(-\frac{k_0}{2} \frac{x^2 + y^2 + \delta^2}{z_R + iz}\right) \sum_{\sigma=\{-1,1\}} (\sin\alpha + i\sigma\cos\alpha) \exp\left(-\frac{k_0}{2} \frac{-2\sigma D y}{z_R + iz}\right) \\
&\approx \exp\left(-\frac{k_0}{2} \frac{x^2 + (y - i\cot\alpha D)^2}{z_R + iz}\right),
\end{aligned} \tag{S4}$$

where the last line is obtained by applying Taylor series $\exp(x) \approx 1 + x$ for $x \ll 1$ twice. Then the intensity of the postselected beam at $x = 0$ is

$$|\psi_{\text{postselected}}(x = 0, y)|^2 = \exp\left(-\frac{k_0}{2} \frac{z_R}{z_R^2 + z^2} \left((y - \mathrm{Re}(\tilde{D}))^2 - \mathrm{Im}(\tilde{D})^2 - \frac{2z}{z_R} \mathrm{Im}(\tilde{D})(y - \mathrm{Re}(\tilde{D}))\right)\right), \tag{S5}$$

where $\tilde{D} = -i\cot\alpha D$. Maximum of the intensity lies at

$$\begin{aligned}
y &= F\mathrm{Im}(\tilde{D}) + \mathrm{Re}(\tilde{D}) \\
&= F\mathrm{Re}(D)\cot\alpha - \mathrm{Im}(D)\cot\alpha,
\end{aligned} \tag{S6}$$

where the second term in the right-hand side of the last line is negligible when $F \gg 1$. Then we obtain Eq. 2 in the main manuscript.

S3 Proof of the equivalence of Fig. 2a and c

This section justifies the replacement of the convex lens and free-space of its twice focal length with the concave lens (equivalence of Fig. 2a and c in the main manuscript). We prove that a Gaussian after a concave lens with a focal length of f is equivalent to a Gaussian that passes a convex lens with the same focal length and then propagates by the distance of $2f$. We use the general expression of a Gaussian below:

$$\psi(x, y; z_R, z) = \exp\left[-\frac{k_0}{2} \frac{x^2 + y^2}{z_R + iz}\right], \tag{S7}$$

where k_0 is the wave number, $z_R = k_0 w_0^2/2$ is a Rayleigh length, w_0 is the beam waist, and z is the propagation distance from the Gaussian focus. The phase profile of a lens that has a focal length

of f follows

$$\phi(x,y) = \pm k_0(\sqrt{x^2+y^2+f^2} - f), \tag{S8}$$

where + and − correspond to the concave and convex lens, respectively. By applying the Taylor expansion

$$\sqrt{x^2+y^2+f^2} \approx f + (x^2+y^2)/2f, \tag{S9}$$

which is true at $\sqrt{x^2+y^2} \ll f$, Eq. S8 can be simplified to

$$\phi(x,y) = \pm i\frac{k_0}{2}\frac{x^2+y^2}{f}. \tag{S10}$$

Then the transmitted beam is

$$\begin{aligned}\psi_t &= \exp\Big[-\frac{k_0}{2}\frac{x^2+y^2}{z_R+iz}\Big]\exp\Big[\pm i\frac{k_0}{2}\frac{x^2+y^2}{f}\Big] \\ &= \exp\Big[-\frac{k_0}{2}\frac{x^2+y^2}{\frac{z_R}{1+(z_R/f)^2} \pm i\frac{z_R^2/f}{1+(z_R/f)^2}}\Big] \\ &= \psi(x,y;\frac{z_R}{1+(z_R/f)^2}, \pm\frac{z_R^2/f}{1+(z_R/f)^2)}).\end{aligned} \tag{S11}$$

The Gaussian right after a concave lens can be represented as

$$\psi(x,y;\frac{z_R}{1+(z_R/f)^2}, \frac{z_R^2/f}{1+(z_R/f)^2)}), \tag{S12}$$

while the Gaussian after the convex lens and free-space propagation of $2f$ is

$$\psi(x,y;\frac{z_R}{1+(z_R/f)^2}, -\frac{z_R^2/f}{1+(z_R/f)^2)} + 2f). \tag{S13}$$

Comparison between Eqs. S12 and S13 shows straightforwardly that the only difference is the large arguments, which is approximately same when $(z_R/f)^2 \gg 1$. Given that z_R is the Rayleigh length of the collimated beam from the source, the assumption is true.
